# The Impact of Social Cognition on the Real-Life of People with Epilepsy

**DOI:** 10.3390/brainsci11070877

**Published:** 2021-06-30

**Authors:** Arminas Jasionis, Kristijonas Puteikis, Rūta Mameniškienė

**Affiliations:** 1 Center for Neurology, Vilnius University, 08661 Vilnius, Lithuania; ruta.mameniskiene@santa.lt; 2 Faculty of Medicine, Vilnius University, 03101 Vilnius, Lithuania; kristijonas.puteikis@mf.stud.vu.lt

**Keywords:** emotion recognition, generalized epilepsy, quality of life, social cognition, strange stories, temporal lobe epilepsy, theory of mind

## Abstract

**Background.** Previous research has demonstrated the impairment of social cognition (SC) in people with epilepsy. It is associated with worse social functioning and quality of life; however, the influence on real-life outcomes is unknown. The purpose of this study was to investigate how SC is associated with epilepsy variables and real-life outcomes (education, employment and relationships) among patients with epilepsy (PWE). **Methods.** Eighty-one PWE completed tasks of theory of mind (ToM) (faux pas recognition (FPRT) and Happé Strange Stories test (HST)) and emotion recognition (ER) (Reading of the Mind in the Eyes (RMET)). Variables reflecting their education, employment and relationship status were treated as endpoints in search of association with SC. Data from a matched group (n = 30) of healthy controls (HCs) were used for comparison of ToM abilities. **Results.** ToM scores were lower among PWE as compared to HCs (U = 1816.0, *p* < 0.0001 (HST), U = 1564.5, *p* = 0.020 (FPRT)). All SC tests were associated with the level of education (OR = 1.22, 95% confidence interval (CI) = 1.09 to 1.36 (RMET), OR = 1.20, 95% CI = 1.02 to 1.40 (HST), OR = 0.93, 95% CI = 0.87 to 1.00 (FPRT)). The results of ToM and ER testing were not associated with employment (χ^2^ = 33.423, *p* < 0.0001) if adjusted for the level of education (B = 0.804, OR = 2.23 (95% CI = 1.33 to 3.76), *p* = 0.002). SC abilities did not differ between PWE who were single and those in a relationship (U = 858.5, *p* = 0.541 (HST)), t= −1.236, *p* = 0.220 (RMET), U = 909.5, *p* = 0.271 (FPRT)). Conclusion. Better social cognition skills are linked to a higher level of education among PWE. SC probably has less influence on professional achievements and interpersonal relationships.

## 1. Introduction

The ability to interact with people in an appropriate, meaningful and rewarding way is necessary to build relationships, engage in societal activities and discover work opportunities. The conceptual definition of epilepsy encompasses the fact that besides the impact of epilepsy on cognitive, mental and physical health, people with epilepsy (PWE) may endure social consequences associated with their condition [1]. Overall, PWE are observed to have difficulties in establishing social networks and relationships and may have lower rates of employment—such findings are probably multifactorial and can be attributed to changes in brain function, psychological comorbidities and external determinants (e.g., social stigma) [2]. The capacity to understand thoughts, feelings and the behavior of other individuals can be defined as social cognition (SC) and regarded as an objectively measurable cognitive domain. Measures of social cognition include tests of emotion recognition (ER) and the assessment of theory of mind (ToM), concepts that are closely related. While results concerning genetic generalized epilepsies (GGE) remain limited, certain deficits of ER and ToM are observed among PWE with temporal lobe (TLE) and extratemporal (ETE) epilepsies [3,4,5,6,7,8].

Research of SC in adult PWE has been centered around the relationship of ToM or ER and clinical variables as well as the association between SC and other cognitive domains. Measures of quality of life and social functioning in the context of SC have also been explored, but the possible influence of worse SC on real-life outcomes (e.g., being employed or having a partner) has not been reported [7,8]. The analysis of objectively identifiable features eliminates the risk that SC deficits might lead to the under- or overestimation of one’s social and professional circumstances, as investigated through subjective scales [2,9].

To provide new viewpoints of SC in epilepsy, we enrolled patients with different types of epilepsy and examined their ability with regard to mentalization and ER. The aims of this study were (1) to compare the performance of patients with TLE, ETE, GGE and healthy controls (HCs) in ToM tasks, (2) to describe the association between epilepsy variables and SC and (3) to determine whether emotion recognition (ER) and ToM are associated with the quality of life, level of education, employment and being in a relationship.

## 2. Materials and Methods

### 2.1. Study Sample

A cross-sectional study was performed at the Epilepsy Center of Vilnius University Hospital Santaros Klinikos between December 2019 and August 2020. Eligible adults with epilepsy and a stable antiseizure medication (ASM) regimen were invited to participate in the study during routine clinical visits. We excluded those with confirmed psychiatric or neurodevelopmental conditions (e.g., depression, autism spectrum disorder, intellectual disability), drug or alcohol abuse, brain trauma or sensory/motor deficits that would evidently prevent the comprehension and completion of cognitive tasks. By excluding persons with only overt forms of autism, we investigated whether clinically significant deficits in ER and ToM in people with epilepsy could be detected even in those who do not already have a formal diagnosis of autism spectrum disorder.

The study was approved by the Vilnius Regional Bioethics Committee (approval no. 2019/12-1173-661) and conducted in accordance with the principles of the WMA Declaration of Helsinki. All eligible patients signed an informed consent form before entering the study. Participation in ToM tasks was offered for healthy hospital visitors with no history of neurological, psychiatric or other chronic disorder or concomitant use of medications. The group was targeted to match epilepsy patients by age, sex and education.

### 2.2. Clinical and Socioeconomic Data

Clinical variables (seizure types and frequency, ASMs), EEG and neuroimaging data were collected from medical records. The National Hospital Seizure Severity Scale (NHS-3) was used to evaluate seizure severity (the result attributed to the most severe seizure type was used for analysis) [10]. The Neurological Disorders Depression Inventory in Epilepsy (NDDI-E) served as a measure of depressive symptoms and was selected as a potential confounder alongside clinical and neuropsychological data in regression models [11]. The quality of life of PWE was assessed by the Lithuanian version of the 31-item Quality of Life in Epilepsy inventory (QOLIE-31) [12]. We used the total score and the subscores of emotional well-being, energy/fatigue, cognitive function, medication effects, overall quality of life, seizure worry, and social function for further analysis.

All participants were asked to provide information about their employment status and were ascribed to one of the categories from the International Standard Classification of Occupations (ISCO-08) [13]. They indicated the years spent in the educational system and the level of education achieved, which was matched to a category from the 2011 International Standard Classification of Education (ISCED) [14]. Finally, patients reported their relationship (married, single, widowed, divorced, having a partner) and family (number of children) status.

### 2.3. Tests of Social Cognition

Three cognitive tests were used to measure the patients’ ability to understand human emotion and behavior. The Lithuanian version of the Reading of the Mind in the Eyes test (RMET) included 36 eye photos and the participant was asked to select one of four emotions that best describes mental state of a person in the photo (each correct answer was scored as one point) [15]. The faux pas recognition test (FPRT) consisted of six stories that described situations which included socially inappropriate behavior (e.g., an unintentional remark which may hurt the feelings of the other person) [16]. The FPRT was scored based on responses to six questions, addressing different aspects of the patient’s understanding of the story: detecting faux pas (two questions), understanding inappropriateness, recognizing the character’s intentions, beliefs, and being able to empathize. Two control questions were used to judge whether the patient understood the content of the story (if not, the story was not scored for faux pas). Moreover, the FPRT included three control stories with no faux pas (the result was treated as a separate measure). The Happé strange stories test (HST) comprised eight situations, in which the participant had to detect a lie, white lie, bluffing, persuasion or misunderstanding presented in different social situations [17,18]. Each story was scored on a two-point scale, depending on the patient’s ability to interpret the situation. The tests of social cognition had been adapted and validated for the Lithuanian population prior to the current study [19].

### 2.4. Data Analysis

The targeted sample size was based on a calculation for a one-way analysis of variance (ANOVA) test that would include 3 groups (TLE, ETE and GGE) with at least 22 patients in each and achieve a power of 0.8 (α = 0.05, f = 0.40).

All data were analyzed in IBM SPSS v26. Chi square (χ^2^) and Fisher’s exact tests were used for categorical data. Mann–Whitney U, Kruskal Wallis H tests and Spearman’s correlation were employed for the analysis of ordinal or non-normally distributed variables (as evaluated by Shapiro–Wilk or Kolmogorov–Smirnov tests). The Student’s t test and one-way ANOVA served to describe normally distributed data.

Binary logistic regression was used in search of variables associated with achieving higher education, being employed, and having a partner. Ordinal logistic regression was applied to explore relationships between tests of SC and the level of achieved education on the ISCED scale. A multiple linear regression model was used in search of QOLIE-31-related variables. The multivariable models included clinical variables (duration of epilepsy, number of ASMs and seizure frequency) and results of the NDDI-E as potential confounders. R (v4.1.0) was used to create graphs.

## 3. Results

### 3.1. General Findings

Among the participants, 27 had been diagnosed with genetic generalized, 25 with temporal (6 right, 11 left, 8 bilateral TLE) and 29 with extratemporal (9 right, 16 left, 4 bilateral ETE) epilepsy. Patient characteristics are presented in Table 1. Available data from a healthy control group (n = 30, 11 males and 19 females, age Md = 27.5 (range 18–60)) were used to compare the performance in ToM tasks (HST and FPRT).

There was no statistically significant difference in the distribution of sex (χ^2^ = 7.036, *p* = 0.071), years of education (H = 7.044, *p* = 0.071) or achieved level of education (H = 6.695, *p* = 0.082, according to ISCED) among groups of GGE, ETE, TLE and HCs. The age of healthy controls (Md = 27.5 years, range 18 to 60) did not differ from the epilepsy group (Md = 30 years, range 18 to 66) as well (U = 1076.0, *p* = 0.356).

**Figure 1:**
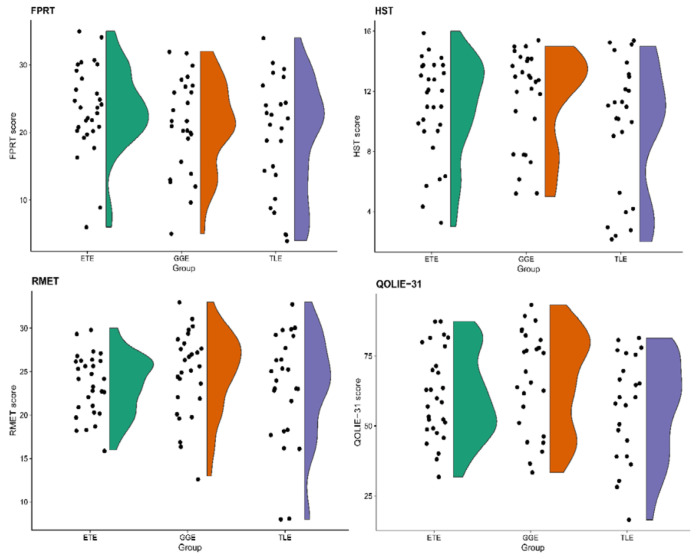
Raincloud plots for tasks of social cognition and quality of life scores among groups of patients with extratemporal (ETE), generalized (GGE) and temporal lobe (TLE) epilepsies. FPRT—faux pas recognition test (total score), HST—the Happé strange stories test, RMET—Reading of the Mind in the Eyes test, QOLIE-31—The 31-item Quality of Life in Epilepsy inventory (total score).

HST scores were higher among HCs as opposed to PWE (U = 1816.0, *p* < 0.0001). While there was no significant difference between HCs and PWE in the number of stories correctly identified to contain faux pas (U = 1104.5, *p* = 0.448) and the ability to detect faux pas in the stories themselves (U = 1157.0, *p* = 0.693), PWE had lower scores across FPRT subscales measuring the understanding of inappropriateness (U = 1511.5, *p* = 0.045), intentions (U = 1941.0, *p* < 0.0001), beliefs (U = 1525.0, *p* = 0.036) empathy (U = 1513.0, *p* = 0.043). The total FPRT score and the ability to recognize control stories among PWE were lower as well (U = 1564.5, *p* = 0.020 and U = 1525.5, *p* = 0.004, respectively). The comparison of FPRT and HST among groups of GGE, ETE, TLE and HCs is presented in Table 2 and Figure 1.

An additional comparison among subgroups of PWE confirmed that the results of the HST (H = 3.187, *p* = 0.203) and the FPRT (F = 2.226, *p* = 0.115) did not differ among patients with TLE, ETE and GGE. The single statistically significant difference emerged from the FPRT question of intention comprehension (H = 8.506, *p* = 0.014, post hoc ETE > TLE (adj. *p* = 0.011)).

Among PWE, the results of cognitive testing were not statistically different between male and female participants (Student’s t = −0.279, *p* = 0.781 (RMET), U = 794.0, *p* = 0.853 (HST) and U = 671.0, *p* = 0.311 (FPRT)) and was not related to age (r_s_ = −0.130, *p* = 0.248 (RMET), r_s_ = −0.128, *p* = 0.253 (HST), r_s_ = −0.069, *p* = 0.539 (FPRT)).

The results of all tests of social cognition did not differ in distribution depending on the etiology of epilepsy (F(2,78) = 0.480, *p* = 0.621 (RMET), H = 2.112, *p* = 0.348 (HST) and H = 0.484, *p* = 0.785 (FPRT)). Non-significant results were present in terms of EEG localization in focal epilepsies (F(2,51) = 1.163, *p* = 0.321 (RMET), H = 3.601, *p* = 0.165 (HST), F(2,51) = 0.611, *p* = 0.547 (FPRT)). However, all tests were associated with the number of ASMs used (r_s_ = −0.262, *p* = 0.018 (RMET), r_s_ = −0.366, *p* = 0.001 (HST), r_s_ = −0.244, *p* = 0.028 (FPRT)). The RMET and HST, but not FPRT scores were associated with the frequency of seizures (r_s_ = −0.222, *p* = 0.046, r_s_ = −0.301, *p* = 0.006 and r_s_ = −0.172, *p* = 0.124, respectively). Only the HST was related to the duration of epilepsy (r_s_ = −0.344, *p* = 0.002). Seizure severity was associated with the number of control stories detected (r_s_ = −0.261, *p* = 0.020) in the FPRT as well as the test’s faux pas detection (r_s_ = −0.257, *p* = 0.021) and understanding of inappropriateness (r_s_ = −0.220, *p* = 0.049) subscores. There was a weak correlation between neuropsychological test scores and the NDDI-E (r_s_ = −0.239, *p* = 0.035 (RMET), r_s_ = −0.274, *p* = 0.015 (HST), r_s_ = −0.276, *p* = 0.014 (FPRT)).

### 3.2. Social Cognition and Education

**Figure 2:**
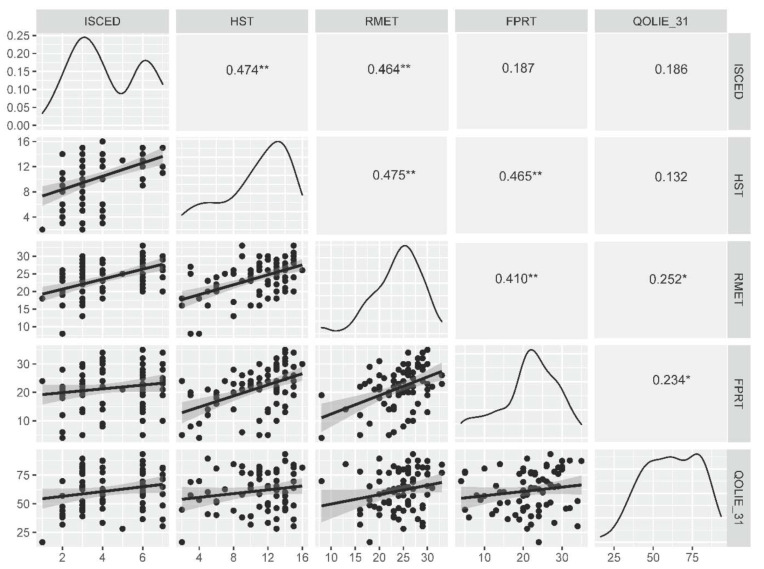
Correlations between the level of education, ToM and ER tasks and the measure of quality of life. *—*p* < 0.05, **—*p* < 0.0001. FPRT—faux pas recognition test (total score), HST—the Happé strange stories test, ISCED—The International Standard Classification of Education, RMET—Reading of the Mind in the Eyes test, QOLIE-31—The 31-item Quality of Life in Epilepsy inventory (total score).

The number of years spent in the education system correlated with HST and RMET scores (r_s_ = 0.450, *p* < 0.0001 and r_s_ = 0.505, *p* < 0.0001, respectively) as well as the number of control stories recognized during the FPRT (r_s_ = 0.288, *p* = 0.010), but not with the total FPRT score (r_s_ = 0.194, *p* = 0.083) or separate FPRT questions (r_s_ < 0.250, *p* > 0.050 for all). The same result was obtained for correlations between social cognition test scores and the ISCED scale (r_s_ = 0.474, *p* < 0.0001 (HST), r_s_ = 0.464, *p* < 0.0001 (RMET), and r_s_ = 0.245, *p* = 0.030 (FPRT control stories)) as presented in Figure 2.

A multivariable ordinal regression model revealed that RMET, HST and FPRT scores are statistically significantly associated with the level of education, as measured on the ISCED scale (Table 3). In a binary regression model with the same set of variables (χ^2^ = 29.388, *p* < 0.0001), the results of tests of social cognition were statistically significant predictors of achieved tertiary education as well (OR = 1.24, 95% CI = 1.05 to 1.48 (RMET), OR = 1.46, 95% CI = 1.12 to 1.89 (HST) and OR = 0.91, 95% CI = 0.83 to 1.00 (FPRT)). Please note that better performance on the FPRT was associated with lower education.

### 3.3. Social Cognition and Employment

While both HST and RMET scores were higher among PWE who are employed (U = 1186.5, *p* < 0.0001 and U = 1134.0, *p* < 0.0001, respectively), only the ability to understand inappropriateness from the FPRT was positively associated with employment (U = 1028.5, *p* = 0.012). Among PWE who were employed (n = 50, 61.7%), the results of social cognitive tests did not differ between those with a job that requires high or low–medium level of skill (ISCO-08 groups 1–3 and 4–9, respectively): U = 213.0, *p* = 0.057 (RMET), U = 213.5, *p* = 0.056 (HST) and Student’s t = 0.081, *p* = 0.936 (FPRT).

The HST score was a statistically significant predictor in a multivariable binary regression model in which employment was the dependent variable (Table 3). However, this association was no longer valid (B = 0.167, OR = 1.18 (95% CI = 0.95 to 1.47), *p* = 0.135) when the level of education (B = 0.804, OR = 2.23 (95% CI = 1.33 to 3.76), *p* = 0.002) was included in the model (χ^2^ = 33.423, *p* < 0.0001).

### 3.4. Relationships and Quality of Life

PWE who were single and those who were either married, in a relationship or widowed performed equally well on the HST (U = 858.5, *p* = 0.541), the RMET (t = −1.236, *p* = 0.220) as well as on the FPRT (U = 909.5, *p* = 0.271) and its separate questions (*p* > 0.05). Respective results were obtained in a binary regression model (χ^2^ = 4.172, *p* = 0.760), in which none of the variables significantly contributed to model fit (OR = 1.01, 95% CI = 0.90 to 1.14 (RMET), OR = 0.96, 95% CI = 0.81 to 1.14 (HST) and OR = 1.03, 95% CI = 0.96 to 1.11 (FPRT)). While the total FPRT score was higher among PWE who had one or two children as compared to those having none (H = 9.011, *p* = 0.029), the post hoc results remained not statistically significant when adjusted for multiple testing.

There was a statistically significant correlation between RMET scores and the total QOLIE-31 score (r_s_ = 0.252, *p* = 0.027) as well as the overall QoL (r_s_ = 0.267, *p* = 0.019) and emotional well-being (r_s_ = 0.288, *p* = 0.011) subscales. The total FPRT score was associated with the total QOLIE-31 score (r_s_ = 0.234, *p* = 0.041), the overall QoL (r_s_ = 0.236, *p* = 0.039) and the medication effects (r_s_ = 0.285, *p* = 0.012) subscores. The FPRT question regarding inappropriateness was related to the QOLIE-31 overall QoL score (r_s_ = 0.237, *p* = 0.038), emotional well-being (r_s_ = 0.240, *p* = 0.036), medication effects (r_s_ = 0.327, *p* = 0.004), social function (r_s_ = 0.231, *p* = 0.043) and the total QOLIE-31 (r_s_ = 0.272, *p* = 0.017) scores. The question concerning the intentions of characters in a FPRT situation correlated with medication effects (r_s_ = 0.267, *p* = 0.019) and overall QoL (r_s_ = 0.264, *p* = 0.021). Being able to detect control stories was related to higher values of the seizure worry and social function scales (r_s_ = 0.230, *p* = 0.045 and r_s_ = 0.241, *p* = 0.036, respectively). No relationship between the HST and QOLIE-31 results was detected (r_s_ < 0.25, *p* > 0.05). The results of a multiple regression model with the QOLIE-31 total score as the dependent variable are presented in Table 4.

## 4. Discussion

### 4.1. Social Cognition and Epilepsy

Our findings confirmed reports from other study groups that PWE, especially persons with TLE or ETE, perform worse on ToM tasks when compared to HCs [7,8,20,21]. The results of post hoc analyses in the current study suggest that the difference between PWE and HC is mostly driven by worse performance in patients with focal epilepsies. However, the capacity to perform SC tasks was similar among patients with TLE, ETE and GGE, suggesting that some impairment is present in GGE as well. While more evidence regarding ER and ToM among patients with GGE is needed, deficits in these cognitive domains are supported by other controlled studies [3]. However, differences in SC among patients with focal epilepsies and generalized epilepsies are rarely examined. Similarly to the findings in our study, one report found no difference in empathy and ER tasks between TLE and GGE, and another presented post hoc results revealing worse performance among PWE with focal epilepsies as opposed to HC or patients with GGE [4,6]. Overall, no difference is found in facial emotion perception between the two epilepsy subgroups [6,22,23,24]. Investigations of SC in focal epilepsies alone are more frequent—authors of a recent review of 42 studies concluded that no significant variation exists between patients with TLE and ETE in ER and ToM tasks, which is in line with our findings [25].

We could not state that ToM and ER tasks are associated with age or sex, but they were related to epilepsy variables, such as seizure frequency or the number of ASMs. This finding might represent a burden of ASMs or frequent ictal events on subtle social cognitive functions, in a similar way as is true for other cognitive domains (e.g., attention, memory, etc.) [26]. It may be especially relevant for the HST, as it was linked to both seizure frequency and the duration of epilepsy. The matter requires further investigation, but the results of this ToM task may be more influenced by impairments in other cognitive domains (as compared to the FPRT) [27]. On the other hand, HST could reflect those ToM functions that form early in life alongside other cognitive domains and are more prone to damage by epileptic activity.

### 4.2. Real-Life Outcomes in the Context of Social Cognition

Most SC studies in epilepsy assessed quality of life as an outcome which could depend on ToM and ER. The resulting findings hinted that social cognition may impact self-perceived quality of life: ToM tasks have been associated with the WHO QOL-100 scale and the QOLIE-89, but not QOLIE-31 [21,24,28,29,30]. While we saw a correlation between SC and QOLIE-31, it is important to note that a negative relationship was detected between measures of depressive symptoms and tasks of social cognition. The subsequent inclusion of NDDIE-E scores in a regression analysis revealed that neither ToM, nor ER are significant predictors of global quality of life. Therefore, the direct influence of social cognition on subjective measures of QoL might be only slight, with mood disorders having greater impact. A report by Wang et al. addressed the relationship between the Social and Occupational Functioning Scale for Epilepsy (SOFSE) and the FPRT among patients with TLE [8]. Our hypothesis was that a similar result may be replicated while (1) expanding the study sample to include patients with ETE and GGE as well as TLE and (2) focusing on factual real-life outcomes rather than subjective scales.

#### 4.2.1. Social Cognition and Education

When performed, the analysis of formal education and ToM yields conflicting results. A recent study in the healthy population provided evidence that ToM (measured as FPRT scores) is not associated with age or education [31]. This is supported by our findings, which indicate no clear correlation between either age or education and the FPRT; the relationship even swayed towards being inverse in a multivariable regression model. It was argued that the ToM may be acquired outside the bounds of the education system (i.e., through other everyday stimuli) [31]. However, schooling was found to be associated with better FPRT performance in studies of TLE [7,20]. In the current study, a relationship between the level of achieved education and the ability to understand the Happé strange stories and recognize emotions from pictures was also present (even if adjusted for seizure variables and depressive symptoms). As the directionality of this association is questionable, the latter may reflect that either (1) ToM and ER ensure sustained participation in academic activities or (2) these cognitive domains require an academic environment to fully develop in adult PWE. Earlier studies stated that ToM abilities develop in early childhood; however, more recent research proposes that adolescence is crucial for further development of advanced ToM [32].

The reasons why the HST, but not the FPRT, was associated with the level of education remain unknown. Both tests are similar tools for the detection of subtle ToM deficits, which are regarded to be independent from any co-existing cognitive impairment [7,20,33]. However, such domain specificity was mostly proven for the FPRT. One explanation could be that the FPRT was more difficult than the HST for the participants—this is supported by HST scores approaching ceiling levels [34,35]. That is, the HST (along with the RMET) might reflect a less advanced level of ToM that is essential for productive functioning in society. The impaired perception of nonliteral communication in the HST could, in turn, indicate wider cognitive deficits and even some lack of introspection (given that the HST was not associated with the results of QOLIE-31) [2,36].

#### 4.2.2. Social Cognition and Employment

As one’s chances of being employed depend on education; the associations between having a job and results of ToM and ER tasks were shown to be confounded by the inclusion of the level of education in the regression model. In this way, measures of social cognition were presumably related to employment mostly because of their link with academic milestones. Interestingly, measures of social cognition were similar among PWE who are employed, regardless of them having either highly or low–medium skilled employment. Therefore, ToM and ER are probably important abilities across a wide spectrum of work environments. It may also be speculated that current tools to measure SC have questionable ecological validity among adult PWE, as they present situations that are relatively simple in comparison to real-world scenarios. Successful employment could require skills in a higher order of ToM. Although alternative methods, including measures of empathy and sarcasm, and visual or video tasks were proposed to delineate ToM abilities, the question of how they represent real-life functioning remains open [37].

#### 4.2.3. Social Cognition and Relationships

In our sample, none of the SC tasks were associated with being married or in a relationship. This finding suggests that good performance in ToM and ER tasks is not essential to be in an affectionate relationship. Impaired ToM means that an individual may encounter difficulties in understanding thoughts or feelings of his/her partner. However, it does not imply the inability to recognize them after more time and effort, which are required in all successful relationships. For emotional connections, SC may be less significant than in formal circumstances, where time and willingness to interpret the cognitive states of others is limited.

In addition, the office-based testing of SC might not fully reveal the individual’s potential of mentalizing in a romantic setting. For instance, one study demonstrated that the primes of a loved one (in contrast to neutral primes) can activate love-related networks and enhance the performance in RMET [38]. This suggests that, while being in a romantic relationship, SC may function better than in non-sexually bonded relationships. Maintaining relationships is equally important and, although ToM is associated with a partner’s well-being, one study of mentalization among older adults showed that ToM abilities are more related to friendships, but not relationships with relatives [39,40].

Nevertheless, building and maintaining romantic relationships is overall complicated for PWE—seizures, psychiatric comorbidities and perceived stigma are probably more important determinants than SC for such interpersonal difficulties [41,42].

### 4.3. Study Limitations

The limitations of this study consist of its cross-sectional design and the inclusion of relatively small subgroups of different types of epilepsy. While the aggregation of results from a heterogeneous sample that included patients with TLE, ETE as well as GGE is substantiated by similar demographic variables and comparable performance during testing of SC between these subgroups, the findings should be replicated in larger patient groups of each epilepsy type. The results of correlation analysis and subgroup comparisons should be interpreted with caution because of multiple statistical testing.

Given that our analysis did not include measures of other cognitive functions or global intellect, the impact of SC on real-life outcomes was presented solely in the context of epilepsy variables and depressive symptoms. Moreover, only the patients with overt autism spectrum disorder (diagnosis confirmed by a psychiatrist) were not included in the present study. As autistic traits were not evaluated, it is possible that at least some of the participants had mild forms of autism spectrum disorder. It is therefore important to note that lower skills of SC could have resulted from influences of subclinical comorbidities in addition to pathogenetic events of epileptogenesis.

## 5. Conclusions

Our study provides further evidence that PWE perform worse on ToM tasks than healthy individuals. However, we could not observe differences in the results of ToM and ER between subgroups of GGE, ETE and TLE. SC was observed to be associated with seizure frequency and the number of ASMs, but not with the etiology of epilepsy or seizure lateralization. While there was a correlation between SC and quality of life, it may be biased by coexisting symptoms of depression. Finally, ToM and ER were associated with the level of education a patient has achieved, but not directly with the chances of being employed or establishing intimate relationship. Further research is needed to investigate how SC might influence the opportunities for PWE to lead a fulfilling life in a society.

## Figures and Tables

**Table 1:** Clinical and demographic characteristics of people with epilepsy enrolled in the study.

	GGE (n = 27)	ETE (n = 29)	TLE (n = 25)	Test Value	Significance
	n (%), Median (Range) or Mean (SD)				
**Male/female**	5 (18.5%)/22 (81.5%)	13 (44.8%)/16 (55.2%)	13 (52.0%)/12 (48.0%)	χ^2^ = 6.981	0.030 *
**Age (years)**	26 (18–42)	30 (19–66)	36 (23–57)	*H* = 13.635	0.001 *
**Years with epilepsy**	11.67 (7.67)	16.86 (9.27)	16.92 (10.89)	F = 2.823	0.066
**ASMs**				*H* = 8.261	0.016 *
**None**	3 (11.1%)	1 (3.4%)	0		
1	14 (51.9%)	8 (27.6%)	10 (40.0%)		
2	6 (22.2%)	8 (27.6%)	5 (20.0%)		
3	3 (11.1%)	8 (27.6%)	3 (12.0%)		
≥4	1 (3.7%)	4 (13.7%)	7 (28.0%)		
**Etiology**				χ^2^ = 73.378	<0.0001 **
Genetic	27 (100%)	1 (3.4%)	2 (8.0%)		
Structural		8 (27.6%)	12 (48.0%)		
Unknown		20 (69.0%)	11 (44.0%)		
**MRI lesion**	2, 7.4% (0 seizure-related)	11, 37.9% (7 seizure-related)	18, 72% (17 seizure-related)	32.543	<0.0001 **
**Experiences GTCS**	25 (92.6%)	20 (69.0%)	16 (64.0%)	χ^2^ = 6.685	0.035 *
GTCS frequency ^1^	2 (1–3)	2 (1–5)	1.5 (1–5)	*H* = 0.054	0.974
GTCS severity	15.04 (2.85)	15.40 (4.48)	14.14 (3.90)	F = 0.484	0.619
**Experiences other seizures than GTCS**	17 (63.0%)	24 (82.8%)	24 (96.0%)	χ^2^ = 9.118	0.010 *
Non-GTCS frequency ^1^	3 (1–5)	3 (2–5)	3 (1–5)	*H* = 2.465	0.292
Non-GTCS severity	1 (1–7)	6 (1–12)	4 (1–12)	*H* = 17.929	<0.0001 **
**NDDI-E**	9 (6–20)	12 (6–20)	11 (6–24)	*H* = 4.034	0.133
**Years in the education system**	14 (9–17)	13 (9–22)	15 (6–20)	*H* = 6.038	0.049 *
**ISCED**	4 (2–6)	3 (2–7)	5 (1–7)	*H* = 4.886	0.087
**Tertiary education**	12 (44.4%)	5 (17.2%)	13 (52.0%)	χ^2^ = 7.909	0.019 *
**Is employed**	16 (59.3%)	17 (58.6%)	17 (68.0%)	χ^2^ = 0.604	0.739
**ISCO 1–3 (high level of skill)**	10 (62.5%)	5 (29.4%)	12 (70.6%)	χ^2^ = 6.486	0.039 *
**Has a partner/widowed**	17 (63.0%)	13 (44.8%)	13 (54.2%)	χ^2^ = 1.852	0.396
**Partner status**				7.997	0.409
Single	8 (29.6%)	13 (44.8%)	10 (40.0%)		
Married	8 (29.6%)	9 (31.0%)	10 (40.0%)		
In a relationship	9 (33.3%)	3 (10.3%)	3 (12.0%)		
Divorced	2 (7.4%)	3 (10.3%)	1 (4.0%)		
Widow	0	1 (3.4%)	0		
Unknown	0	0	1 (4.0%)		
**Children**				5.828	0.407
0	16 (59.3%)	18 (62.1%)	14 (56.0%)		
1	6 (22.2%)	7 (24.1%)	2 (8.0%)		
2	5 (18.5%)	3 (10.3%)	7 (28.0%)		
3	0	1 (3.4%)	1 (4.0%)		
Unknown	0	0	1 (4.0%)		

^1^—1—less than once/year, 2—once per year, 3—once per month, 4—once per week, 5—every day, *—*p* < 0.05, **—*p* < 0.0001.

**Table 2:** The comparison of social cognitive tests and quality of life between groups of people with epilepsy and healthy controls.

	GGE (n = 27)	ETE (n = 29)	TLE (n = 25)	HC (n = 30)	Test Value	Significance
	Median (Range) or Mean (SD)					
**Faux pas recognition test**						
Number of faux pas stories detected	5 (1–6)	5 (2–6)	5 (1–6)	5 (2–6)	*H* = 3.621	0.305
Number of control stories detected ^1^	3 (0–3)	3 (0–3)	3 (0–3)	3 (2–3)	*H* = 8.339	0.040 * HC > TLE/ETE/GGE ^2^
Faux pas detection in the stories	10 (2–12)	10 (4–12)	9 (2–12)	10 (4–12)	*H* = 2.993	0.393
Understanding Inappropriateness	4 (0–6)	4 (0–6)	4 (0–6)	5 (2–6)	*H* = 5.635	0.131
Intentions	2 (0–5)	2 (0–5)	1 (0–4)	4 (0–6)	*H* = 30.890	<0.0001 * HC > TLE/ETE/GGE
Belief	3 (0–5)	3 (1–6)	3 (0–6)	4 (0–6)	*H* = 6.506	0.089
Empathy	4 (1–6)	4 (1–6)	4 (0–6)	5 (1–6)	*H* = 6.771	0.080
Faux pas total score	21.19 (6.87)	23.45 (6.47)	19.24 (8.66)	25.20 (7.98)	F = 3.284	0.024 * HC > TLE
**Happé strange stories test**	13 (5–15)	12 (3–16)	11 (2–15)	14 (10–16)	*H* = 18.997	<0.0001 * HC > ETE/TLE
**Reading of the Mind in the Eyes test**	24.85 (4.83)	23.48 (3.491)	23.04 (6.45)	n/t	F = 0.946	0.393
**Quality of life** ^3^						
Seizure worry	49.98 (31.04)	53.15 (27.84)	43.20 (29.06)	n/a	F = 0.745	0.478
Overall QoL	77.5 (32.5–100)	66.25 (15–90)	60 (32.5–82.5)	n/a	*H* = 6.181	0.045 * GGE > TLE
Emotional well-Being	68.65 (22.10)	60.68 (18.39)	58.61 (22.58)	n/a	F = 1.615	0.206
Energy/Fatigue	55.77 (20.82)	53.63 (16.93)	47.93 (23.56)	n/a	F = 0.951	0.391
Cognitive	61.43 (24.40)	61.32 (19.78)	52.52 (22.59)	n/a	F = 1.275	0.286
Medication effects	66.02 (24.59)	71.32 (19.73)	61.23 (28.65)	n/a	F = 1.099	0.339
Social function	87.5 (24–100)	66 (13–100)	77 (0–100)	n/a	*H* = 2.214	0.331
QOLIE-31 total score	65.78 (18.10)	61.03 (15.85)	56.73 (18.61)	n/a	F = 1.642	0.201

^1^—n = 2 (1.8% of PWE + HC) missing, ^2^—significant only before Bonferonni correction, ^3^—n = 4 (4.9% of PWE) missing, n/t—not tested, n/a—not applicable, *—*p* < 0.05.

**Table 3:** Regression analysis for real-life outcomes represented by the level of achieved education, according to the ISCED scale, and the binary fact of being employed at the moment of cognitive testing (variables with *p* < 0.05 are bold), ^1^—missing n = 3 (3.7%), ^2^—χ^2^ = 34.697, *p* < 0.0001, ^3^—χ^2^ = 21.148, *p* = 0.004, constant B = −3.105, *p* = 0.149, OR = 0.45 (95% CI = 0.00–3.03).

	ISCED						Employment					
Independent Variable	Univariable Models			Multivariable Model ^1,2^			Univariable Models			Multivariable Model ^1,3^		
	B Coefficient	*p* Value	OR (95% CI)	B Coefficient	*p* Value	OR (95% CI)	B Coefficient	*p* Value	OR (95% CI)	B Coefficient	*p* Value	OR (95% CI)
Duration of epilepsy	−0.370	0.099	0.96 (0.92 to 1.01)	0.003	0.895	1.00 (0.96 to 1.05)	−0.019	0.422	0.98 (0.94 to 1.03)	0.040	0.229	1.04 (0.98 to 1.11)
Number of ASMs	**−0.436**	**0.009**	**0.65 (0.47 to 0.90)**	−0.213	0.327	0.81 (0.53 to 1.24)	**−0.505**	**0.009**	**0.60 (0.41 to 0.88)**	−0.093	0.752	0.91 (0.51 to 1.62)
Seizure frequency	−0.266	0.113	0.77 (0.55 to 1.07)	0.053	0.800	1.06 (0.70 to 1.59)	**−0.548**	**0.010**	**0.58 (0.38 to 0.88)**	−0.309	0.296	0.73 (0.41 to 1.31)
NDDI-E	**−0.141**	**0.008**	**0.87 (0.78 to 0.96)**	−0.090	0.101	0.91 (0.82 to 1.02)	−0.099	0.095	0.91 (0.81 to 1.02)	−0.024	0.739	0.98 (0.85 to 1.12)
RMET	**0.199**	**<0.0001**	**1.22 (1.12 to 1.33)**	**0.196**	**<0.0001**	**1.22 (1.09 to 1.36)**	**0.178**	**0.002**	**1.20 (1.07 to 1.34)**	0.104	0.138	1.11 (0.97 to 1.27)
HST	**0.249**	**<0.0001**	**1.28 (1.14 to 1.44)**	**0.180**	**0.025**	**1.20 (1.02 to 1.40)**	**0.282**	**<0.0001**	**1.33 (1.14 to 1.54)**	**0.260**	**0.011**	**1.30 (1.06 to 1.58)**
FPRT	0.040	0.130	1.04 (0.99 to 1.10)	**−0.070**	**0.044**	**0.93 (0.87 to 1.00)**	0.050	0.112	1.05 (0.99 to 1.12)	−0.033	0.481	0.97 (0.88 to 1.06)

**Table 4:** A multiple regression model with QOLIE-31 total score as the dependent variable (F(7,68) = 14.180, *p* < 0.0001, Adjusted R^2^ = 0.552, missing n = 5 (6.2%)). Variables with *p* < 0.05 are bold.

Independent Variable	B Coefficient	*p* Value
Duration of epilepsy	0.072	0.407
Number of ASMs	−0.123	0.226
Seizure frequency	−0.171	0.062
NDDI-E	**−0.678**	**<0.0001**
RMET	0.123	0.191
HST	−0.193	0.067
FPRT	0.010	0.916
Constant	102.866	**<0.0001**

## Data Availability

Raw study data is available from the authors upon reasonable request.
